# Influenza pandemic planning for cancer patients

**DOI:** 10.3390/curroncol13040012

**Published:** 2006-08

**Authors:** P.M. Battershill

**Keywords:** Influenza, pandemic, planning

## Abstract

Given that an influenza pandemic is likely within the next few years, the World Health Organization has recommended that policymakers take action to mitigate the consequences of such a pandemic. Because of the increased risk of patients with cancer developing complications of influenza, policymakers in cancer care should immediately begin planning for changes to resource allocation, clinical care, and the consent process during a pandemic.

## 1. INTRODUCTION

The World Health Organization (who) continues to report new cases of human infection with the H5N1 avian influenza virus in Turkey and Southeast Asia. The next influenza pandemic may not involve the H5N1 strain, but given past epidemiologic patterns, emergence of a new pandemic strain in the near future is likely. The who has recommended that policymakers take action to mitigate the consequences of such an event 1. National, regional, and institutional plans have been developed, but I am aware of none that have addressed the unique needs of patients with cancer.

Cancer patients are at increased risk of developing complications of influenza—specifically, bacterial superinfection. Patients with cancer face all the risks of the general population during a pandemic and also have increased susceptibility to infection as a result of their illness. For example, myeloma and chronic lymphocytic leukemia are frequently associated with hypogammaglobulinemia; patients with these diseases are therefore at risk for bacterial infections. Increased susceptibility can also result from the general debility caused by cancer. Additionally, many cancer treatments increase the risk of complications of influenza through myelosuppression. These unique risks mean that policymakers in cancer care must urgently develop plans for changes in resource allocation, clinical practice, and consent procedures during an influenza pandemic.

## 2. DISCUSSION

### 2.1 Resource Allocation

It is estimated that 15%–35% of the general population will become clinically ill during an influenza pandemic 2. The percentage of health care workers becoming ill could be higher than that of the general population because of occupational exposure to the virus. Preparedness plans therefore prioritize health care workers for influenza vaccination and prophylaxis with antiviral medications. Despite these interventions, many health care workers will be unavailable because of illness, fear of occupational exposure to influenza, and illness within their own families. With a reduced workforce, a reduction in the number of cancer treatments delivered is an inevitability. Thus, rationing of oncology services will be a reality.

We will have to make difficult decisions if the workforce is reduced to 85% of normal. At 65% of normal staffing, the decisions about who will receive treatment will be agonizing. Clearly we will strive to deliver potentially curative treatments. Not so obvious is how we will manage palliative therapies. We may need to modify our concept of futility. For example, should we give first- and second-line, but not third-line, metastatic chemotherapy treatments? Patients unable to receive chemotherapy in such circumstances will still need expert supportive care; palliative services will, however, be similarly strained during a pandemic.

Issues of distributive justice will be front and centre in the discussions of how we treat cancer patients during a pandemic. Perhaps we should give preference to therapies that are less labour-intensive, allowing us to treat more patients. For example, in the adjuvant treatment of early breast cancer, the use of doxorubicin and cyclophosphamide (ac chemotherapy) for 4 cycles may allow us to treat more patients than would be possible if we used chemotherapy regimens requiring 6 or 8 cycles. The ac chemotherapy may be slightly less effective in preventing recurrence of the cancer in any one individual, but if more people can be treated, the less effective treatment may be justified.

Cancer care providers should assess their current service capacity. Institutions currently functioning near capacity will have greater difficulty providing service when staff levels are decreased. Clearly, these institutions will have greater challenges in resource allocation.

### 2.2 Changes to Clinical Practice

In addition to standard measures of influenza prevention—vaccination, careful attention to hand washing, and antiviral prophylaxis for people at high risk—changes to standard oncology practice may lessen the risks to cancer patients. Health care providers must always balance the risks of harm with the potential for benefit when making treatment recommendations.

During a pandemic, the potential for benefit with chemotherapy would be unchanged, but the risk of harm would be increased to a degree that cannot be readily quantified. Neutropenic patients who contract influenza will be at significantly increased risk of harm from bacterial superinfection. The use of hematopoietic colony–stimulating factors to prevent chemotherapy-induced neutropenia is not routine with most chemotherapy protocols 3. Greater use of such factors to prevent neutropenia may be reasonable during a pandemic, but would increase the cost of treatment at a time when health care budgets will be severely strained. Increased use of prophylactic antibiotics may prevent some superinfections 4, but at the cost of increased bacterial resistance and antibiotic related diarrhea.

Many patients will not be able to afford the extra costs of the colony-stimulating factors and antibiotics, raising the possibility that an influenza pandemic would worsen the already existing health disparities based on socioeconomic status. To ensure a just distribution of resources, planners should incorporate an ethical component into their planning as suggested in the report by the Joint Centre for Bioethics at the University of Toronto 5. The suggested framework identifies key ethical issues and provides a 15-point guide for ethical influenza pandemic planning.

### 2.3 Consent Issues

Cancer patients need to know that myelosuppressive treatment could carry greater risk during a pandemic. In addition to the concerns mentioned earlier, the usual supports for patients with febrile neutropenia will be severely limited. Access to hospital beds will be limited both by increased demand and by staff illness. In this circumstance, patients may well make an informed choice for a potentially less efficacious but less myelosuppressive treatment.

## 3. CONCLUSIONS

Policymakers in cancer care should immediately start planning for cancer care delivery during an influenza pandemic. However, no single plan will suffice for all oncology practices. The needs of a pediatric service will differ from the needs of a bone-marrow transplant service.

We have the opportunity now to prepare for cancer care provision during a pandemic. To assure optimal treatment and fair distribution of resources, this planning should start immediately.
